# The Importance of Mimicking Dermal-Epidermal Junction for Skin Tissue Engineering: A Review

**DOI:** 10.3390/bioengineering8110148

**Published:** 2021-10-20

**Authors:** Mina Aleemardani, Michael Zivojin Trikić, Nicola Helen Green, Frederik Claeyssens

**Affiliations:** 1 Biomaterials and Tissue Engineering Group, Department of Materials Science and Engineering, Kroto Research Institute, The University of Sheffield, Sheffield S3 7HQ, UK; maleemardani1@sheffield.ac.uk (M.A.); m.trikic@sheffield.ac.uk (M.Z.T.); n.h.green@sheffield.ac.uk (N.H.G.); 2 Insigneo Institute for in Silico Medicine, The Pam Liversidge Building, Sir Robert Hadfield Building, Mappin Street, Sheffield S1 3JD, UK

**Keywords:** dermal-epidermal junction, skin tissue engineering, scaffolds, physical factors, topographical features

## Abstract

There is a distinct boundary between the dermis and epidermis in the human skin called the basement membrane, a dense collagen network that creates undulations of the dermal–epidermal junction (DEJ). The DEJ plays multiple roles in skin homeostasis and function, namely, enhancing the adhesion and physical interlock of the layers, creating niches for epidermal stem cells, regulating the cellular microenvironment, and providing a physical boundary layer between fibroblasts and keratinocytes. However, the primary role of the DEJ has been determined as skin integrity; there are still aspects of it that are poorly investigated. Tissue engineering (TE) has evolved promising skin regeneration strategies and already developed TE scaffolds for clinical use. However, the currently available skin TE equivalents neglect to replicate the DEJ anatomical structures. The emergent ability to produce increasingly complex scaffolds for skin TE will enable the development of closer physical and physiological mimics to natural skin; it also allows researchers to study the DEJ effect on cell function. Few studies have created patterned substrates that could mimic the human DEJ to explore their significance. Here, we first review the DEJ roles and then critically discuss the TE strategies to create the DEJ undulating structure and their effects. New approaches in this field could be instrumental for improving bioengineered skin substitutes, creating 3D engineered skin, identifying pathological mechanisms, and producing and screening drugs.

## 1. Introduction

**Figure 1:**
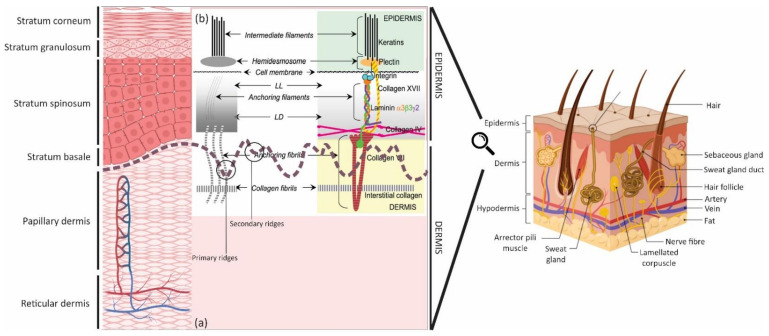
Skin schematic: (**a**) microstructure of the skin layers, magnifying the epidermis and dermis; created by Biorender and (**b**) DEJ representation. Schematic of different anatomical substructures, seen by transmission electron microscopy (the left diagram) (LL: lamina lucida and LD: lamina densa) and depicting the identified molecular components of substructures (the right diagram); reused with permission [30]—Reproduced by permission of The Royal Society of Chemistry.

Skin is the largest and one of the most dynamic organs in the human body. There are many studies related to the skin since it is the first semi-permeable barrier between the body and the environment [1,2]. Despite the current broad knowledge about skin, still, its function is not fully understood. It consists of three layers (Figure 1); the first two layers, the dermis and epidermis, play an essential role in protection, and the meeting point of these two layers is known as the dermal-epidermal junction (DEJ) [3,4]. Skin regeneration occurs throughout life, and the whole epidermis renews itself every 28 days. However, its ability for repair is limited to full-thickness loss smaller than 4 cm in diameter and is age-related [5,6]. Understanding the regeneration process thoroughly can lead to new treatments for large wounds, skin diseases, and new drugs to promote regeneration [7,8]. The aim of tissue engineering (TE) is to create constructs that recapitulate the distinctive features of native skin, and advances have been made, which have developed TE skin constructs either for repairing full-thickness skin defects or modelling wound healing or other pathological conditions [9,10].

However, these constructs lack some of the anatomical and physiological features of the skin. Since the skin’s functions depend on its 3D anatomical structure, research in this field is quite significant [11,12]. Current bioengineered skin substitutes and models often have a flat interface design at the DEJ [13,14,15]. In native skin, the DEJ structure is corrugated, forming rete ridges (50–400 µm in width) in the epidermis projecting more deeply into the dermis and generating dermal papillae (50–200 µm in depth) where the dermis comes closest to the skin surface [16]. DEJ has two significant roles, maintenance of structural integrity and control of the cellular microenvironment, which are essential for the appropriate keratinocytes functioning within these areas. The keratinocyte stem cell niches are located at the DEJ, and the topography of this structure is extremely significant to maintain tissue structure and mechanical properties, as well as directing critical processes of wound healing [17,18]. These structures result in the enhancement of hemidesmosome (HD) numbers at the DEJ, which increase the interface strength (Figure 1b) [19]. HDs are microscopic stud-like structures placed in the epidermal keratinocytes cell membrane and attach these cells to the basement membrane (BM). It is noteworthy that the dimensions of rete ridges vary with body site and age (DEJ flattening) and enhance (rete ridges lengthening) when inflammatory skin diseases such as psoriasis occur [20,21]. There are even skin diseases such as epidermolysis bullosa cases where there is no evidence of rete ridges in the skin structure, resulting in a very loose DEJ and blisters forming even under minor stress [22]. Another example of DEJ changes due to skin diseases is cancer that damages DEJ, which leads to integrity loss [23]. Moreover, the palms of the hands and bottom of the feet experience high friction or shear stress; here, there are more rete ridges, which are deeper and narrower than areas with lower friction, such as the scalp [24]. Furthermore, through applying mechanical forces, the rete ridges enlarge and trigger a rapid proliferation of basal precursors, resulting in the basal layer’s growth. This mechanism preserves the epidermal organization and enhances the regenerative potential of expanded skin [25,26].

The 3D cellular microniches or microenvironments at the DEJ are thought to be the stem cell (SC) niches in the skin, embedding in rete ridge areas with the spatial structure of keratinocyte markers in order to proliferate and differentiate terminally [27,28]. Up to now, several particular factors for regulating keratinocyte differentiation and maintaining stemness have been investigated. For instance, biophysical parameters, such as oxygen tension and shear stress, can trigger differentiation, and signaling from stromal fibroblasts are also influential [29]. To understand the relation between cellular mechanisms and the 3D microenvironment that direct skin responses, further studies are required. Some groups have developed in vitro models of native skin; however, the effects of topographic and biochemical roles of DEJ have not been elucidated completely [14,15,30]. The cellular behavior of skin within microniches (DEJ) remains poorly understood. Research in this field has much potential, namely for (1) enhancing the bioengineered skin substitutes performance [13,31], (2) developing 3D engineered skin [15,32,33], (3) characterizing pathologies, and (4) producing and screening drugs [10,34,35,36]. This review aims to provide an overview of the DEJ structure and function by discussing its effects on skin regeneration. It will also highlight the conventional and tissue engineering techniques used to keep or develop DEJ to mimic the in vivo skin microenvironment in 3D in vitro skin models.

## 2. Ultrastructure

### 2.1. Epidermis

**Figure 2:**
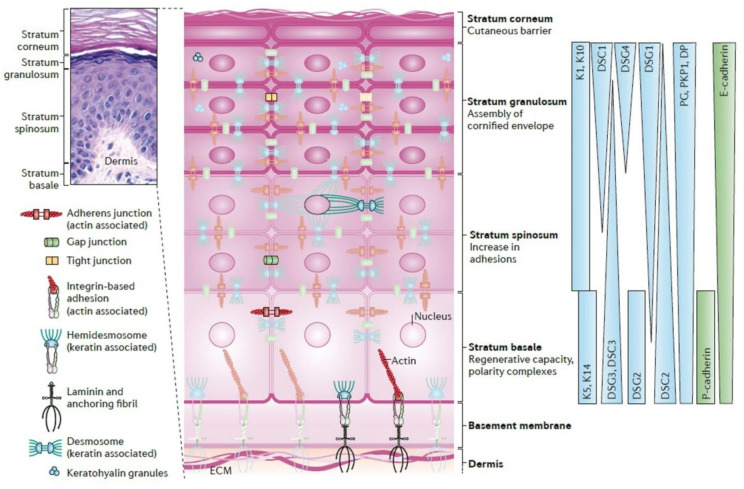
Epidermal architecture. The epidermis has various cellular junctions and cytoskeletal elements; it is ranked one of the most dynamic tissues due to its response to cutaneous damage and ability to regenerate continuously. The asymmetric distributions can be seen in protein expression, signaling activity and cytoarchitectural development within the mature epidermis, depicting the tissue-level polarization and multiple functions. A specific cytoarchitecture is made during keratinocyte differentiation in each layer, including particular cytoskeleton and cell junction types like adherens junctions, tight junctions, desmosomes, and gap junctions. The differentiation-dependent alters the composition and organization of epidermal cytoarchitecture, aiding to drive tissue morphogenesis and the function of each layer. The distribution of specific cytoskeletal and junction components is graded and are also vital to drive morphogenesis. Hematoxylin and eosin (H&E) stained human skin sample illustrates four main layers with varying thickness from 20 to 150 μm and an accompanying schematic. Cytoskeletal and junction components: keratins (Ks), desmogleins (DSGs), cadherins, desmoplakin (DP), desmocollin (DSC), epithelial cadherin (E-cadherin), placental cadherin (P-cadherin), plakoglobin (PG), and plakophilin 1 (PKP1). Reused with permission [44]. Copyright © 2021, Nature Reviews Molecular Cell Biology.

The epidermis is populated by mostly keratinocytes; these cells are potentially the most critical cell population because they generate the stratum corneum, which forms the primary barrier of the skin. This layer is avascular, although it faces constant renewal almost every 28 days through SC, particularly basal layer and hair follicles [16,37,38] (Figure 2). Basal keratinocytes divide to relocate further cell layers; subsequently, the cells develop dense intercellular attachments for mechanical strength, before becoming pyknotic. Pyknosis is the irreversible chromatin condensation in the cell nucleus that is a degenerative condition. The cells thereafter lose their nucleus, and flattening through younger keratinocytes to finally form skin squames in the stratum corneum [39]. This layer is one of the most widely studied cell layers in the body for many reasons. The most probable and significant cause is that it experiences renewal during the whole life provided by available SCs [40,41]. Rete ridges (rete pegs) are extensions of the epidermis, projecting into the underlying connective tissue (dermis) (Figure 1 and Figure 2). They have two essential roles: (1) acting as microniches for epidermal SCs [41,42] and (2) to avoid scar formation; scar tissue lacks rete ridges, which leads to malfunctions such as shearing off more quickly than normal tissue [43].

### 2.2. Dermal-Epidermal Junctions (DEJ)

The zone that joins the epidermis to the dermis encompasses the DEJ (Figure 1 and Figure 2) and contains the cutaneous basement membrane (BM). This region has interdigitated topography that results in an increment in mechanical shear resistance, paracrine diffusion, and surface area between the dermis and epidermis. It also creates microtopographic niches that determine the cellular microenvironments, defining the phenotype and cellular function of keratinocytes [45,46]. This dynamic interface, the DEJ, governs the structural integrity of the skin and provides an operative gap between the sublayers without using any bulk spacer, where the skin sensory receptors are located (within all three layers of skin). Bulk spacers are materials that are used to achieve high sensitivity and output power for sensors [47]. Furthermore, the rete ridges enlarge and trigger a rapid proliferation of basal precursors by applying mechanical forces, resulting in the basal layer’s growth, which results in skin resistance against stresses and pressures [48]. The DEJ controls biomolecule movements between the epidermis and dermis, depending on the molecular size and charge. Under certain circumstances, it also allows migrating and invading cells, both in the normal situation (e.g., melanocytes and Langerhans cells) and pathological conditions (e.g., lymphocytes during the inflammatory response and tumor cells during metastasis). Furthermore, it has essential roles in development, morphogenesis, wound healing, and skin remodeling. Abnormalities of several of these biomolecules have a direct relation to several inherited and acquired DEJ (skin) disorders [49,50].

The DEJ formation commences at 10 weeks of post-fertilization when small undulations of the basal laminae appear, and dermal ridges begin to form as a result of cell proliferation within the basal layer of the epidermis. In the proceeding 5–7 weeks, the primary ridges extend farther into the dermis mature, and sweat glands form. This structure supplies nutrients to the SCs within the suprabasal epidermal layer and creates new skin tissue [51]. There is no longer formation of new dermal ridges after this period. However, at the top of each primary ridge, a downfolding process forms secondary ridges and defines the final shape of the dermal papilla. An inter-ridge dimension is roughly 100–200 µm deep and 70–150 µm wide [52]. As previously mentioned, human dermal and epidermal ridges form during fetal development, although these structures continue to change in adulthood [52] and sometimes due to pathological reasons [53]. At the DEJ, the distribution of cells [54], SCs [55] and biomolecules [48] are not homogenous, and it is not well clarified what differences arise from it.

### 2.3. Dermis

The dermis is located directly beneath the BM, anchored by fibrils and microfibrils (Figure 1). The papillary dermis, the uppermost layer, connects with the epidermis and comprises various fine and loosely arranged collagen fiber. Within this area, there are dermal papillae extensions that act as junctions (DEJ) into the epidermis. The reticular dermis is comprised of dense irregular connective tissue with densely packed collagen fibers [56,57]. The fibrous and nonfibrous dermal matrix can be found in both areas of the dermis; fibrous materials, mainly collagen and elastic fibers, provide tensile strength and flexibility. Nonfibrous materials also act as ground substances to facilitate mass transportation, cellular migration, and preparing a continuous medium [57]. Collagen as a structural component is heterogeneous either in the skin or other parts of the body [58]. Fibroblasts are the predominant cells within the dermis and these cells produce the components of connective tissue [59]. It is a highly dynamic and vascularized layer, which supports epidermis, and at the same time, supplies strength and flexibility to skin (Figure 1) [57,60].

## 3. Proteins

### Proteins and Their Roles in DEJ

In order to transmit force and resist mechanical stress, the DEJ has a vast network of intracellular, transmembrane, and extracellular proteins. The DEJ has a BM that faces the epidermis named the lamina lucida (LL) or lamina rara (electron-lucent zone; 40 nm), while the other side is called the lamina densa (LD; electron-dense zone; ~40 nm). The interfollicular epithelium and skin appendages (containing hair follicles, sweat, and eccrine glands) are lined up by LL and LD (Figure 1b) [30]. Basal epidermal cells are joined to each other through desmosomes, and hemidesmosomes (HDs) connect them to the BM [57] (Figure 2). In addition to the integrin-mediated linkage of cells to the BM, the DEJ also contains anchoring complexes, made of HDs, anchoring filaments, and fibrils (Figure 1 and Figure 2). Within the DEJ, there are several types of protein, including ubiquitous BM components such as collagen (COL) IV, plectin, laminin (Ln), and nidogen in the upper regions, COL IV and VII, anchoring fibrils, and heparan sulphate proteoglycan (mainly in the LD) [49,61]. Several of these DEJ protein components not only act as structural integrators but also play dynamic roles, such as signaling molecules and pathogenic targets.

Two skin cell types (keratinocytes and fibroblasts) contribute protein components of the BM and the DEJ; some are provided by both, while some are made exclusively by one cell type. Basal keratinocytes express HD plaque proteins (e.g., COL IV, V, and VII, Ln 5, and 6 and heparan sulfate proteoglycan), and fibroblasts (within papillary dermis) contribute protein expression at LD (e.g., nidogen, additional Lns, COL IV, and fibronectin) and at sub-basal lamina densa (e.g., COL I, III, and VII) [48,49,61]. There are many reviews on the structure and grouping system of proteins [48,61,62,63,64]. This review covers the essential proteins within the DEJ, and details regarding their characteristics, significance and location are given in Table 1. The exact roles of these proteins in processes, such as regulation, proliferation, survival, or differentiation, are still unclear; therefore, further studies in this field are needed. Since most of the information related to these proteins comes from pathological studies, use of an accurate model system could lead to discovery of basic concepts or new treatments. Ideally, these models should mimic natural characteristics as precisely as possible.

## 4. Stem Cells

Stem cells (SCs) and subsequent progenitor cells mediate skin homeostasis and wound healing are skin stem and progenitor cells in conjunction with circulating cell populations to keep and restore the skin’s integrity and function at rest and after injury. Skin SCs reside within a SC niche that either helps preserve the SCs health and population or supplies cues to regulate their function. As mentioned previously, epidermal SC clusters lie in specific locations relative to the undulations in the DEJ. Physical parameters like topography and physical forces, such as shear forces, cell shape and substrate stiffness, have a critical impact on the balance between SC proliferation and differentiation [65]. The topographical effect at the single-cell level on substrate interactions has been widely investigated; various patterns at the micro- to nano-scale such as grooves, pillars, holes and fibers have been explored [66,67,68]. It has been demonstrated that surface topography directly influences many aspects of cellular behavior, including morphology, spreading, cytoskeleton, motility, and gene regulation. Therefore, it is clear that the DEJ topography regulates SC fate [69].

Further, the scale and dimensions of features are significant determinants of the cellular response. For instance, keratinocytes have been seeded on ECM-coated micro-patterned islands to evaluate the importance of physical parameters on differentiation. Keratinocytes on 20 µm diameter circular islands trigger terminal differentiation, whereas cells on 50 µm islands remained spread, with no differentiation [69,70]. On larger islands, keratinocytes formed a stratified microepidermis with SCs in the basal layer and differentiated cells, characterized involucrin, and transglutaminase 1 expression in the suprabasal layer [70]. Nevertheless, the importance of the DEJ topography on cellular behavior and SCs, in particular, is mainly unexplored. DEJ mimicking could be counted as the engineering of SCs niches since most of the skin stem and progenitor cells are located within DEJ; this engineering is able to lead to SC recruitment, survival, and function, in turn optimizing SC function and expanding SC therapies. Without the formation of a proper SC niche, the survival and function of SCs are likely to be negatively affected, which can lead to a decrease in the effectiveness of clinical outcomes.

### Stem Cells in Epidermis and DEJ

In the stratum corneum, keratinocytes are lost as nonviable, anuclear keratinized squames, sloughed off every day to reduce the chance of detrimental microbial colonization and nullify routine wear and tear. For this reason, skin requires continuous replenishment, and epidermal SCs supply this. Many groups have identified multiple heterogeneous SC populations within the interfollicular epidermis and hair follicles [71]. Generally, SC proliferation is confined to the basal layer attached to a BM; the SC progeny differentiate, detaching the BM to leave the basal layer and go through a specified cellular differentiation program. The basal progenitors are self-renewing and express keratin 5 and 14 markers, giving rise to transit-amplifying (TA) cells and terminally differentiated cells, which express keratin 1 and 10 and involucrin [72]. The generation of the multi-layered interfollicular epidermis, lipid-producing sebaceous glands or firm hair structures is possible through the ability of keratinocytes to differentiate into several cell lineages. The specific lineage selection is principally defined by the cell’s location, indicating the critical role of instructive signals from the microenvironment [73,74].

It has been suggested that the interfollicular epidermis comprises a reservoir of quiescent basal cells, which is predominantly responsible for regeneration, likely compartmentalized around the hair follicles [75]. The reversible state of a cell that does not divide and can re-enter cell proliferation is called quiescence. Some adult SCs in the quiescent state have the ability to activate rapidly under certain circumstances such as injury [76]. Quiescent human interfollicular epidermis SCs, located at the top of rete ridges, express high levels of α6 and β1 integrins and proliferative markers such as Leu-rich repeats and immunoglobulin-like domains 1 (LRIG1) and melanoma-associated chondroitin sulphate proteoglycan (MCSP). High levels of α6 integrin and keratin 15 are observed in the quiescent cells at the bottom of rete ridges, although expression of the latter is influenced by age. It is fascinating that there is the homogeneous expression of keratin 15 throughout the basal layer in infants, while in adults, at the tip of the ridge, the expression is higher [77]. Still, it is controversial to determine the exact location of human quiescent epidermal SCs in the basal layer. The studies show that the quiescent cells have the highest potential to sustain long-term regeneration [55,78]. However, it also has been reported that the formation of a SC niche along a free-moving basal lamina might result in the construction of undulations with accumulating SCs at the tips of fingerlike structures [79].

Two different models describe the proliferation and differentiation patterns of interfollicular epidermal SCs in the mature epidermis [75]. The traditional epidermal proliferative unit (EPU) model describes epidermal keratinocytes as being organized into a column-like structure with two types of basal cells, SC and TA. TA cells continue to differentiate and generate all the suprabasal cells [80,81]. The fact that the basal layer cells are heterogeneous and have high expression levels of integrins support this model [81]. A second model is based on asymmetrical divisions that are found within basal layer cells [82,83]. The SCs divide to generate a self-renewing daughter SC, which differentiates through the asymmetrical distribution of critical factors, whether the division happens laterally or perpendicularly to the BM [81,84]. Even though these two models provide information on the number and location of interfollicular epidermal SCs within the basal layer, the identity of these SCs remains incomplete [85]. Different hypotheses tried to propose more accurate models, although it is still under debate due to the lack of data. Hence, a better understanding of the DEJ could also help identify skin SCs in more details.

During ageing, skin loses its thickness, DEJ (rete ridges, the reservoir for epidermal keratinocytes), dermal fibroblasts, and melanocytes, which results in thinning (atrophy), fragility, delayed wound healing, and dyspigmentation [20,86]. Within aged human skin, HD components like collagen XVII (COLXVII) become downregulated, which can lead to cutaneous fragility [17,87]. Epidermal SCs express COLXVII that physiologically fluctuates by genomic/oxidative stress-induced proteolysis, and as a result, differential expression of COLXVII causes a driving force for cell competition [88,89]. In order to protect the internal organization of the individual cells, multicellular organisms evolved to solve the conflicts by a process named cell competition, which obliterates suboptimal cells from growing tissues through apoptosis [90]. Due to ageing, loss of COLXVII occurs that limits SC and cell competition, leading to HD fragility and SC depletion, adjacent fibroblasts, and melanocytes. Therefore, it can be concluded that evaluating COLXVII is beneficial either as a biomarker for epidermal SCs or as a quality for self-renewal. Additionally, forced maintenance of COLXVII can be applied as an anti-ageing therapy for the skin [88,91]. Moreover, it has been represented that cellular senescence contributes to skin ageing; during that, senescent melanocytes express the senescence marker, p16INK4A, and induce telomere dysfunction, which restricts the proliferation of surrounding cells like basal keratinocytes. Senescent melanocytes also impact keratinocyte function and cause ageing-associated skin thinning, and it is shown these cells act as drivers of skin ageing [92].

## 5. Conventional Techniques to Preserve the DEJ in Clinical Grafts

Skin grafting is one of the most crucial clinical techniques in dermatology and plastic surgery. Skin grafts can be categorized based on a variety of clinical situations like traumatic wounds and burn injuries; however, generally, they are classified as split-thickness or full-thickness grafts [93]. Several approaches have been conducted to preserve the DEJ anatomical features and improve rapid and robust BM formation in clinical grafts. Dermis decellularization followed by cell seeding and then grafting is one of the successful methods used in animal models and human patients. For example, in clinical use, grafting with AlloDerm (a decellularized dermal matrix) resulted in improved graft take, barrier role and functionality [94,95,96]. Compared with Integra, which has a flat DEJ, decellularized dermis preserves the dermal papillae structure and BM proteins. Furthermore, it produces a bilayered graft comprising both rete ridges and a continuous BM through culturing epithelial cells [96]. Nonetheless, it has been reported that after seeding keratinocytes onto decellularized dermis and reconstituting an epidermis, the dermal topography collapses and finally creates false rete ridges [97]. There are other limitations associated with this approach, including the limited availability of human decellularized dermis, lack of control of its composition, and structure and the potential complications correlated with the decellularization process resulting in disease transfer, structural damage and alteration of dermis mechanical properties.

Another strategy to keep the skin microstructure is the CelluTome epidermal micrograft harvesting procedure; it is an autologous option, which removes the superficial epidermal layer, considerably restricting donor site damage and scarring. This is a simplified and automated epidermal harvesting tool that applies heat and suction to produce epidermal micrografts. Experimental work has demonstrated that the epidermal micrografts developed at the DEJ contained migratory basal layer keratinocytes and melanocytes. However, a reduction in some BM proteins (e.g., COL IV) was observed; thus, it may be that the heat and vacuum combination partially remove the BM and its components. Despite some benefits of the procedure including minimal patient discomfort and no donor-site scarring, it has not been widely adopted as a surgical technique [98].

## 6. Tissue Engineering Strategies: From Basic Concepts to Developing a DEJ

TE has gained massive attention since it has the potential to overcome the challenges of 2D and animal studies [99,100]. Numerous studies were conducted through the 2D cell culture of fibroblasts and keratinocytes to investigate human skin, formation, function, and pathology [100]. Despite understanding the basic knowledge of human skin through 2D cell culture, there are essential differences between cells grown in 2D and 3D environment, in their morphologies and cell-cell interactions; as an example, even 2D co-culture cannot result in ordered stratification and keratinization, which limits the formation and maintenance of a mature epithelium [101]. Animal models have also been used as a testing platform. These models show some resemblance to human skin, although different genes and chemogenetics could lead to controversies [100,102]. For instance, the lack of translation to human physiology is the primary concern of applying animal models for drug discovery and screening. About 50% of the drugs that pass animal testing are toxic for humans, and conversely, some might be nontoxic for humans, although they fail in animals [103]. Furthermore, animal models are inappropriate when it comes to reproducing human skin characteristics (e.g., tumors, autoimmune diseases, and drug therapeutic or toxic responses) [104]. For example, the mouse skin lacks rete ridges, resulting in confounding the analysis and misleading results [105]. In order to overcome these challenges, TE 3D models are considered a better approach [106,107]. The European Union introduced a complete ban on animal models for cosmetics testing in 2013, which was a great trigger to focus on producing alternative platforms [36] that could mimic an in vivo-like microenvironment with an in vitro platform [104].

Different studies have tried to develop skin models by considering intrinsic (genetic and epigenetic) and extrinsic (cell-cell, extracellular matrix (ECM) protein, soluble factors interactions and morphology) factors to mimic skin function. However, it is worth mentioning that there is less attention to mimicking the biophysical characteristics, such as precise anatomical structure [10,104,108,109]. To exemplify, Kim et al. fabricated a fully-matured perfusable/vascularized 3D skin model (P/V full-thickness), containing all three layers of skin through a unique cell-printing platform. This study aims to enhance the human skin’s structural complexity through bioprinting, enabling the precise localization of cell types and biomaterials. Despite observing a successful skin maturation, the model lacked an effective DEJ, which can cause some issues in the longer term [10]. In some studies, the DEJ structure has not been fabricated directly, although it has been claimed that the DEJ could be developed over time through grafting of the TE platform. In one study, a human dermis equivalent (HDE) was fabricated. Human dermal fibroblasts were seeded on gelatin porous microcarriers in a dynamic spinner culture (9 days) and then transferred into a maturation chamber/dynamic bioreactor for 6 weeks to produce a disc-shaped tissue construct (1 mm × 5 mm: thickness × diameter). In the second piece of research, an endogenous human skin equivalent (Endo-HSE) was made by seeding and culturing extracted epidermal cells on the HDE (1 week), followed by lifting to the air-liquid (A-L) interface (14 days). The results show the formation of a basic DEJ and improvement of epidermis barrier function [110].

Since 2012, some studies have been carried out to evaluate the DEJ biophysical features on TE skin models or TE skin substitutes. These studies are detailed in Table 2. A variety of manufacturing techniques could be used to create TE scaffolds [111]; however, up to now, the following strategies have been applied for developing DEJ: photolithography, laser structuring, electrospinning, and 3D printing.

### 6.1. Photolithography

**Figure 3:**
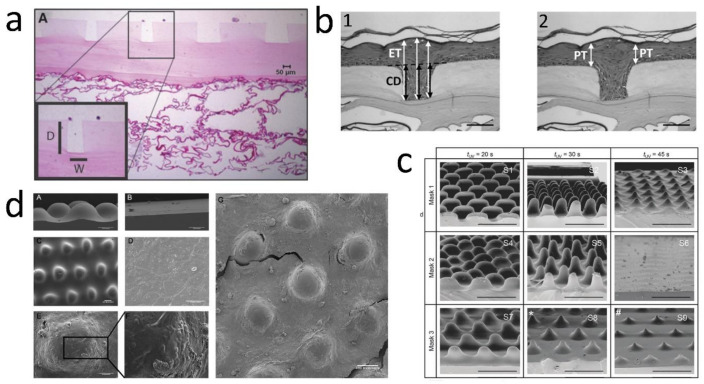
(**a**) µDERM topography (H&E); the insert illustrates the measurements made for depths (D) and widths (W) of the channels. Photo reused with permission [31] © 2012 Tissue Engineering: Part A, Mary Ann Liebert, Inc. (**b**) Morphometric analyses of epidermal thickness in K microniches; (b1) The epidermal thickness (ET) is enhanced in the narrow channels, normalized by the channel depth (CD). (b2) The plateau thickness (PT) was measured immediately adjacent to each channel. Scale bar = 100 µm. Photo reused with permission [13] © 2013 Acta Materialia Inc. Published by Elsevier Ltd. (**c**) SEM images of the patterned substrates fabricated by using 3 different masks. PHEMA molding precursor was exposed to UV light for 3 different intervals. Scale bars: 400 µm. Photo reused with permission [112] © 2015 Integrative Biology, Oxford University Press. (**d**) SEM images of (**A**) patterned, (**B**) flat PDMS substrates before COL coating, (**C**) top view of patterned PDMS substrate before COL coating, (**D**) keratinocytes on flat, and (**E**–**G**) keratinocytes on undulating PDMS substrates. (**F**) A higher magnification view of the boxed region in (**E**). Scale bars: 100 µm and 50 µm (**D**,**E**). Photo reused with permission [113] © 2019 Acta Materialia Inc. Published by Elsevier Ltd.

Photolithographic techniques have been used for many biomedical applications to fabricate microengineered scaffolds [117]. In a study, a two-layer microfabricated dermal-epidermal regeneration matrix (µDERM) was prepared that contained both biophysical (corrugated structure) and biochemical (conjugating fibronectin) signals. This photolithography model (Figure 3a) has studied the effects of the 3D microenvironment (µDERM with 50, 100, 200, and 400 µm-width channels) on epithelialization and basal keratinocyte interactions. It found that the characteristics that closely mimic those in high-friction areas of the body (deep, narrow channels) increase the speed of epithelialization. As an example, at 7 days in this model, 100 µm wide channels generated an epithelium with the same thickness as 50 µm channels after 3 days, although the 200 and 400 µm channels comprised a less-thick epidermis. Furthermore, the expression of β1 integrin was detected and localized in the matrix’s depths [31] demonstrating that the µDERM played a role in keratinocyte proliferation. Continuing the previous study, this group investigated other links between topography and epidermal development, using the µDERM (Figure 3b) as a model to systematically evaluate the impacts of topographical geometries on keratinocyte function. The µDERM dimensions are modified, thickness from 87 µm (beneath channels) to 186 µm (plateaus) and COL–GAG (sponges) to 336 µm thick (Figure 3b). Three distinct functional keratinocyte niches were identified: (1) the proliferative niche (narrow geometries), (2) the BM protein synthesis niche (wide geometries), and (3) the putative keratinocyte SC niche (narrow geometries and corners). Within 50 and 100 µm channels, the epidermal thickness and keratinocyte proliferation were significant, whereas the deposition of Ln 332 was enhanced in 400 µm channels compared to flat surfaces. Interestingly, putative keratinocyte SCs or β1^bri^p63^+^ keratinocytes clusters were observed in channel geometries, which are seen in native skin as well. These results show the importance of µDERM microtopography in designing the TE skin platforms or substitutes [13].

Viswanathan et al. developed a two-step protocol that mimics topographical features of the human DEJ (Figure 3c). The first step created negative patterns by exposing a photocurable formulation to light and controlling the topographical characteristics via the photomask pattern dimensions and UV crosslinking time. The second step translated the negative pattern to the PDMS elastomer, creating substrates with eight unique surface topographies, and primary human keratinocytes were then cultured on these. The biomimetic platform led to cell patterning due to the location of SCs, differentiated cells and proliferating cells [112]. This group also proposed an alternative explanation for the proliferation and differentiation of the keratinocytes, through SC patterning within the DEJ. They suggested that forces from neighboring cells could regulate the keratinocytes behavior depending on the slope of the undulations. By seeding keratinocytes on COL-coated polydimethylsiloxane (PDMS) substrates (Figure 3d) that mimic the DEJ, the SCs become patterned over 24 h organized in a similar way to that seen in the human skin. Cell density and nuclear height were greater at the base of the ridges than at the tips. Interestingly, cells on the tips expressed higher levels of β1 integrin, E-cadherin, desmoglein 3 and F-actin than cells at the base. In contrast, levels of the transcriptional co-factor MRTF-A (MAL) were greater at the base. MAL is one of the signal transduction pathways that regulates keratinocyte differentiation in response to physical cues. Based on the AFM measurements, the Young’s modulus of the cells on the tips was lower than the base. The differences in cell stiffness were dependent on Rho kinase activity and intercellular adhesion [113].

### 6.2. Laser Structuring

**Figure 4:**
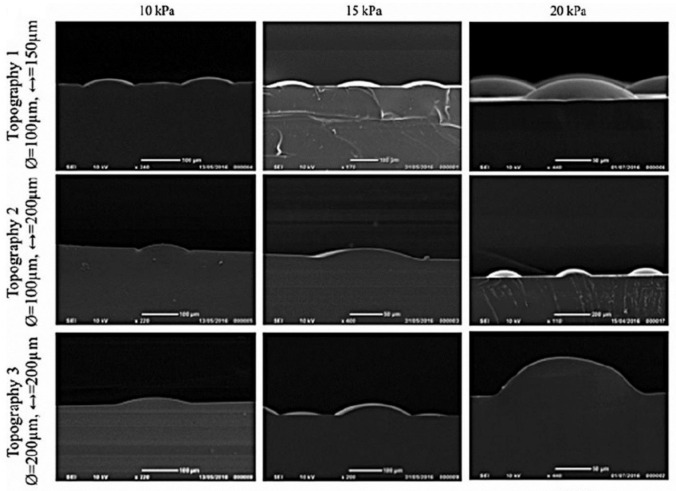
SEM of PDMS stamp depicting PLGA deformation by vacuum pressure. Deformation as a function of topography (1–3) and vacuum pressure (10, 15, or 20 kPa) are investigated. Photo reused with permission [114] © 2019 Tissue Engineering: Part A, Mary Ann Liebert, Inc.

Various laser structuring techniques have been used to create 3D micro/nanofabrication of scaffolds for biomedical applications [118]. With the help of the laser, the undulations produced either directly within the substrate or within the template used to make the structure. Continuing previous studies (mentioned in Section 6.1. Photolithography ), the Viswanathan et al. also created a dynamic model to study how patterning occurs over time. COL-coated poly(d,l-lactide-co-glycolide) (PLGA) membrane was placed over a polyimide sheet containing circular holes of varying in diameter and spacing, made by a drilling laser with no adverse effect on cell viability. A vacuum was applied to create the DEJ’s undulations within the membranes. The heights of the structures were variable depending on the applied pressure and the hole size (Figure 4). Within 48 h of applying the vacuum, cells clustering with high levels of β1 integrin and a SC marker were observed at the bases of the undulations. Furthermore, the clustering of cells with high E-cadherin and nuclear YAP expression was noted, although there was no clustering of differentiated involucrin-positive cells. It has been found that the Rho family of proteins participate during this process since the inhibition of Rho-kinase resulted in the loss of clustering [114].

### 6.3. Electrospinning

Another approach to developing new prototype epidermal-like layers, including pseudo-rete ridge structures, was through electrospun microfabricated scaffolds [14,115,119]. Electrospinning is a well-known technique in biomedicine, with the potential to mimic the ECM structure [120].

#### 6.3.1. Electrospinning on Templates Designed by Stereolithography

**Figure 5:**
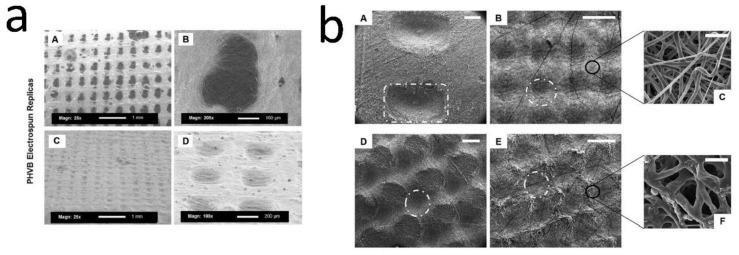
(**a**) Example of the electrospun membrane replicas for both square and rectangular patterns (images **A**–**D**). Photo reused with permission [14] © 2018 Journal of Tissue Engineering, SAGE JOURNALS. (**b**) SEM images of electrospun membranes with three different patterns: (**A**) Pattern A, (**B**) Pattern B, and (**C**) Pattern C (scale bar = 500 μm). (**D**) Air plasma treatment affected the topography of electrospun membranes, scaffolds made with pattern B. Magnifications of specimens with pattern B before (**E**) and after (**F**) air plasma treatment (scale bar = 10 μm). Photo reused with permission [115] © 2021, American Chemical Society.

A reusable template made of polyetheneglycol diacrylate was created by stereolithography, then poly(3-hydroxybutyrate-co-3-hydroxyvalerate) was electrospun over the template to form DEJ microfeatures, and keratinocytes were cultured on it. The platforms ranged from 200 to 1000 µm, edge to edge, with depths varying from 200 to 500 µm (Figure 5a). Cells locate preferably on the microfabricated scaffolds within niche-like areas. Furthermore, increased metabolic activity was observed when keratinocytes were seeded on the structured scaffolds, indicating increased cell proliferation [14]. Patterned polycaprolactone (PCL) electrospun membranes (Pattern A: 192 µm-height and 959 µm-width, Pattern B: 133 µm-height and 360 µm-width and Pattern C: 167 µm-height and 430 µm-width) have been fabricated using microstereolithography-based templates made of methyl methacrylate polymer (RS-F2-GPGR-04) (Figure 5b) and air plasma treated to increase surface functionality. The microfeatures within the membranes enhanced cell metabolic activity and resulted in the accumulation of fibroblasts and keratinocytes at the bottom of these features. The pattern with the smallest feature height and width (Pattern B) led to development of an in vitro DEJ-like skin model that expressed epithelial markers including COL IV and integrin β1 [115].

#### 6.3.2. Laser Structuring of Electrospun Mats

**Figure 6:**
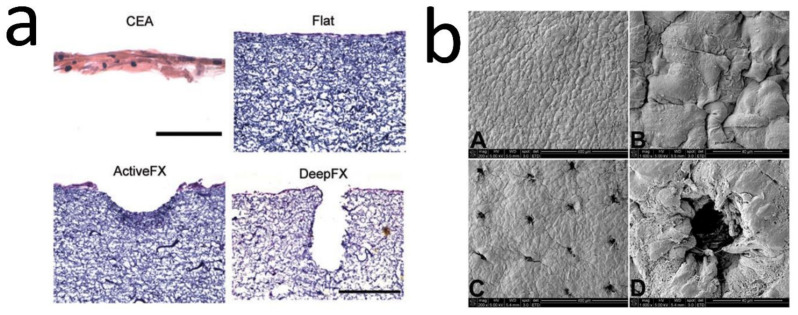
(**a**) H&E of CEAs (at in vitro culture day 20), and flat electrospun COL dermal templates (5 days after seeding with human dermal Fs), ActiveFX & DeepFX (laser-treated groups). CEA and dermal template scale bar = 100 and 200 µm. Photo reused with permission [33] © 2020 Tissue Engineering: Part A, Mary Ann Liebert, Inc. (**b**) SEM of HFs-seeded dermal components before (flat, **A**,**B**) and after (ridged, **C**,**D**) laser treatment, before seeding HKs. Photo used with permission [15] © 2020 Acta Materialia Inc. Published by Elsevier Ltd.

A combination of electrospinning and laser structuring has also been applied in order to create the DEJ undulations. For example, Malara et al. developed a dermal template with stable dermal papillae through electrospinning COL and seeding with human dermal fibroblasts. Laser ablation was used to pattern the cell-seeded dermal fibroblasts, and two templates with either wide and shallow (ActiveFX) or narrow and deep (DeepFX) wells were created (Figure 6a) and grafted to immunodeficient mice for 4 weeks. Ridged templates resulted in rete ridge formation 2 weeks after grafting. In addition, enhanced epidermal thickness and an increase in cell proliferation and stemness were observed [33]. To investigate this in more depth, this group used HFs-seeded dermal templates that had been pre-treated with a CO_2_ laser, creating consistently spaced wells at the surface (Figure 6b). The constructs were seeded with keratinocytes, cultured for 10 days, and grafted onto athymic mice for four weeks. At the grafting time, the rete-ridge structures were observed in the samples and were maintained in vivo. The results were consistent with the previous study, including improved barrier function, increased keratinocyte proliferation, epidermal area, and BM length; also, expression of epidermal SC markers was observed [15].

### 6.4. Additive Manufacturing and Bioprinting

**Figure 7:**
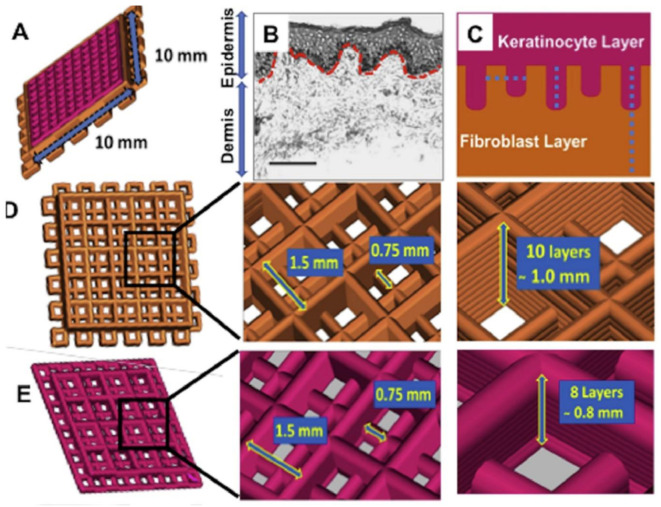
Schematic representation of the design of 3D Bioprinted construct. (**A**) Graphical representation of the Computer-Aided Design of the dual-layered skin model. The epidermis has been designed to create the dermis at regular intervals to form rete ridges. (**B**) Human skin representation, showing the dermis DEJ. (**C**) The structure design strategy for the 10 mm × 10 mm. (**D**,**E**) Detailed layer design dimensions of the dermal and epidermal layers. Design dimensions of the dermal layer (10 layers) and epidermal (8 layered filaments, arranged perpendicular to each other (in X and Y axes) with an interfilament spacing of 0.75 mm and Z-axis increment of 0.08 mm between each layer). Photo used with permission [116] © 2020 Bioprinting. Published by Elsevier Ltd.

Additive manufacturing enables the fabrication of complex geometrical structures, and it has a wide range of applications. In biomedicine, it has gained much attention for the printing of biomaterials for tissue and organ substitutes. Bioprinting is also an additive manufacturing process where bioink, a combination of biomaterials with cells and biomolecules, is used [107]. To replicate anatomically relevant features of DEJ, 3D bioprinting has also been used. It has been applied to fabricate a human cell-based full-thickness skin model, which possesses anatomically relevant structural, mechanical and biochemical characteristics (Figure 7). In this study, 10 mm × 10 mm 3D constructs were printed following an intricate design detailed in Figure 6D,E. The created undulations resulted in significant keratinocyte migration and differentiation, which could indicate successful re-epithelialization. The design and the silk bioink also triggered deposition of BM at the interface; further, the expression of differentiation and cornification markers in a region-specific manner was promoted. Notably, the skin model also resembled native human skin, with several pathways related to skin development and physiology identified, promoting skin development, keratinization, COL fibril, and ECM organization [116].

**Figure 8:**
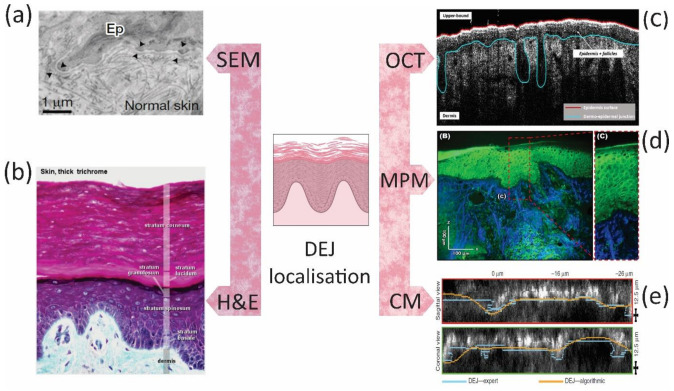
Techniques that are regularly used for the DEJ localization. (**a**) SEM of a normal DEJ (70-nm-thick human skin sections). Photo reprinted with permission [123] © 2017 Macmillan Publishers Limited, part of Springer Nature. (**b**) H&E of a normal skin to identify the epidermal and dermal layers and cells. Photo reprinted from lab.anhb.uwa.edu.au. (**c**) OCT to evaluate skin layer boundaries. Photo reprinted with permission [124] © 2020 Frontiers in Medicine and Dermatology, Frontiers. (**d**) The cross-sectional MPM of the epidermis, DEJ, and dermis. Photo reprinted with permission [125] © 2019 Frontiers in Medicine and Dermatology, Frontiers. (**e**) CM stack of 40 images, 1 μm depth spacing, of dark skin, indicating epidermis, DEJ, and papillary dermis. Photo reprinted with permission [126] © 2015 Journal of Investigative Dermatology, Elsevier.

As highlighted in this review, there are difficulties with using conventional techniques to replicate the heterogeneous native skin. Biologically relevant human skin models would offer tremendous potential for TE and for screening drugs, pathological identification, and understanding the complex physiological processes through bridging the gap between conventional monolayer or 3D cultures and animal models. Current models still have some way to go in recreating the intricate, complex, and multiscale architecture of human skin. For instance, it may be necessary to replicate the different DEJ topographies at different skin sites, since this might be important in skin regeneration and hemostasis, but to date this has not been investigated. However, it is common to analyze the DEJ topography clinically to assess the skin condition or disease [121,122]. Some commonly used techniques for localization of DEJ include scanning electron microscopy (SEM), transmission electron microscopy (TEM), histological staining techniques, optical coherence tomography (OCT), multiphoton microscopy (MPM), and confocal microscopy (CM) (Figure 8). SEM, TEM, and histology (H&E in particular) are commonly used in the laboratory to evaluate the function of developed TE skin platform or scaffold, whereas OCT, MPM, and CM are extensively used in clinics [123,124,125,126]. These techniques provide precise details of DEJ morphology, which could be used to develop personalized, site-specific TE skin substitutes.

## 7. Conclusions and Future Perspective

**Figure 9:**
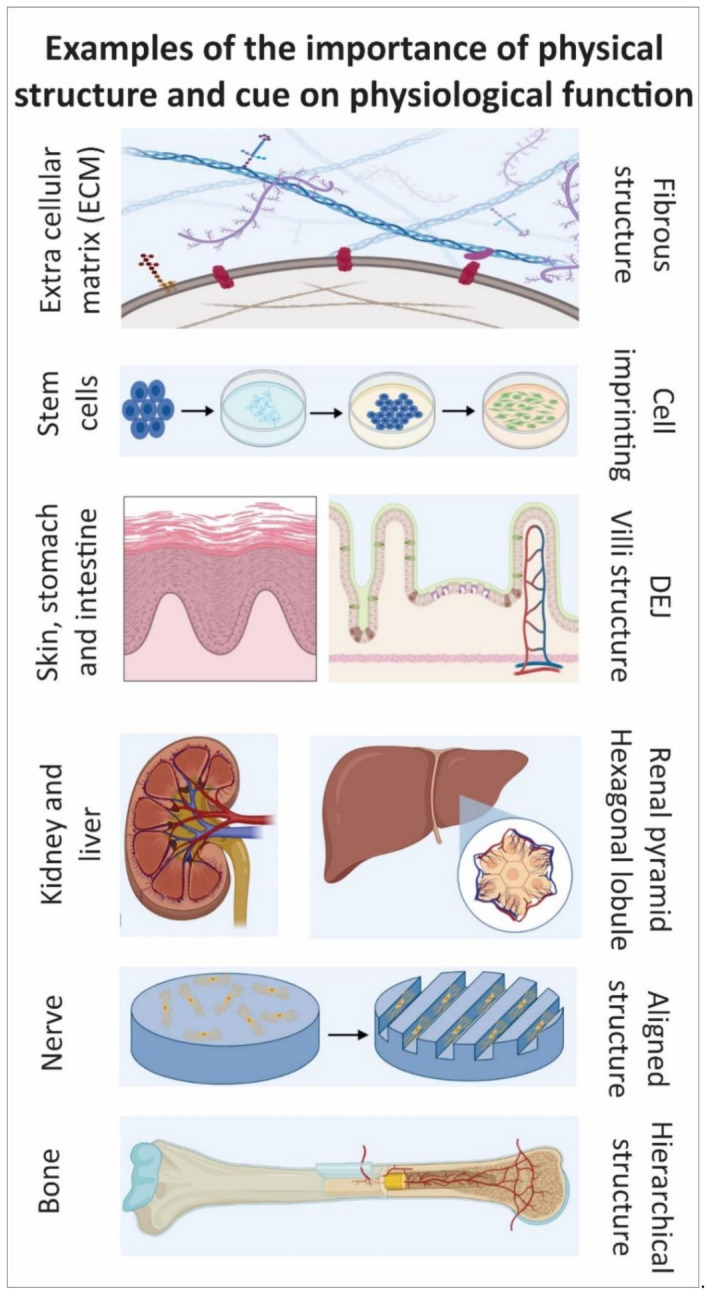
Graphical conclusion—The importance of physical structure on physiological function.

Each tissue or organ has specific geometrical structures such as bone, nerve, liver, and kidney that together with other essential biological, chemical, and physical parameters influence the tissue or organ function. Even at the cellular scale (e.g., ECM structure and cell morphology), the structure and morphology significantly impact function (e.g., biomolecules secretion and cell differentiation). To this aim, micro/nanofabrication techniques have been used to develop constructs that mimic the topographies for tissue regeneration. Many studies have introduced complexity within these constructs to provide topographical cues that are functionally effective. Within the skin, particular physical characteristics are embedded in the DEJ. The DEJ plays a pivotal role in dermal-epidermal homeostasis and adhesion, although it has frequently been omitted from 3D tissue-engineered skins. Therefore, it is not surprising that there are limited studies within this field. However, it is accepted that biophysical factors like topography affect the behavior of cells. The DEJ also creates an important stem cell niche and its structure influences BM formation, epidermal proliferation, differentiation, and stemness. Investigating the DEJ and its roles will lead to a better understanding of skin and the fabrication of more accurate TE skin models and substitutes. An important point to note is that so far although attempts have been made to mimic the overall dimensions of the DEJ within the constructs, the exact geometrical details of undulations have not yet been fully replicated. Additionally, although it has been suggested that epidermal SCs are clustered on the DEJ’s tips, the reason behind this has not been fully characterized. Furthermore, there is incomplete understanding of the key roles of the DEJ in the segregation and promotion of keratinocyte niches, and further research is necessary. This review focuses on the importance of DEJ and introduces potential applications such as developing skin models/platforms for skin substitutes, pathological studies, drug screening, and personalized medicine (Figure 9).

## Figures and Tables

**Table 1:** The list of functional and essential proteins is responsible for the DEJ structural and signaling network. **Abbreviations**: IF, Intermediate filament; HD, Hemidesmosome; AF, Anchoring filament; AP, Anchoring plaques; AFib, Anchoring fibrils; BPAG, Bullous pemphigoid antigen; LL, Lamina lucida; LD, Lamina densa; HF, Hair follicle; Ks, Keratinocytes; Fs, Fibroblasts.

Name and Type			M_W_ (kDa)	Located at	Main Function	Characteristics
IF	Keratins (cytokeratins)	1 & 10	50–100	Suprabasal layers	• Formation of the backbone of the IF network connecting to HDs • Connecting to desmosomes • Providing an extensive mechanical framework	• Express by basal Ks (Interfollicular epidermis)
		5 & 14	50 & 58	Basal layers	• Attaching intracellular cytoskeleton to HD	• Express by basal Ks
HD	HD components	HD1*	450–500	Inner HD plaque	• Anchoring epidermis steadily to LD • Attaching keratin filaments to the basolateral epidermal surface	• Plectin molecule • Associating with cytoskeleton • Distributed throughout epidermis (stratified squamous epithelium)
		HD2	230	Plasma membrane of basal Ks		• Identical to BPAG1
		HD3	200			• Correspond to β4 (subunit of α6β4 integrin)
		HD4	180			• Identical to BPAG2
		HD5	120			• Correspond to α6 (subunit of α6β4 integrin)
	Dystonin (BP230 or BPAG1*)		230	Inner HD plaque	• Attaching intracellular cytoskeleton to HD (cell-matrix adhesion by keratins)	• Intracellular non-COL protein
	BPAG2		180	HD complex	• Facilitating HD assembly through aiding to cluster BPAG1 and plectin Not fully known	• COL transmembrane protein; extracellular domain • COL-like repeats: GXY, X represents any amino acid, known as COL XVII (COL17A1) • Anchoring complex within the LL
	Integrin	β4 & α6	205 & 160		• Cell-matrix or cell-cell adhesion • Transducing signals to regulate gene expression and cell growth	• Transmembrane glycoprotein receptors • Heterodimeric molecules • Genetically distinct α and β subunits
	Integrin: α and β polypeptides complexes	α2β1		Lateral surface of basal Ks	• Cell-cell interactions	• Ligand-binding
		α3β1		Both locations of α2β1 & α6β4 & exclusively in the mature epithelium	• Contributing to basal Ks anchoring to BM (specific to epithelial structures) • Keeping DEJ integrity	• Ligand-binding Ligand: Ln α3 chain (located within Ln 5 & 6 complex)
		α6β4		Ventral surface (opposed to BM zone)	• Cell-matrix stable adhesion (basal Ks to BM)	• Ligand-binding • Promoting the assembly of stable anchoring contacts
		αv: αvβ5, αvβ6 & αvβ8		αvβ5 & αvβ8: adult epidermis (very low levels). αvβ6: SCs in the HF & in Ks in culture	• αvβ5 & αvβ8: Binding mainly to vitronectin. • αvβ6: Binding mainly to fibronectin & hyperproliferation under circumstances	• Binding to RGD motifs
AF	Laminin (Ln)	5; subunits: α3, β3 & γ2	190/165, 140& 155/105	LL, surround sweat, eccrine glands & hair follicles	• BM assembly, connecting HD from LD (primary link between HD integrin α6β4 & LD of BM) • InterHD BM formation • Focal adhesions	• BM glycoproteins • Thin & threadlike structures (2–4 nm diameter) • Secreted by Ks • Results from truncation of all 3 constituent chains • Encoded by the genes LAMA3, LAMB3 & LAMC2 • Binding directly with the COL XVII & amino-terminal end (NC1) domain of COL VII
		6; Subunits: α3, β1 & γ1		Within DEJ	• Regulating cellular adhesion & migration (differently from Ln 332) • Dictating the response of epithelial cells to mechanical stimulation	• Formation: Ln 5 associate with intracellular Ln β1γ1 dimer • Product of Ks & other epithelial cells, particularly amniotic epithelium
		10; subunits: α5, β1 & γ1		Interfollicular epidermis & blood vessels in the dermis	• Promoting the proliferation and migration of epidermal Ks • Maintaining the dermal papilla • Regulating the T cells level Not fully known	• Product of human dermal microvascular endothelial cells
AP	Collagen (COL)	IV (mostly α5 & α6)	540 (trimer)	Mostly in LD, sweat glands & blood vessels	• Network-forming COL • Forming the backbone of BM	• Synthesized by both Ks & Fs
AFib	COL	VII	900 (trimer)	LD & extend into dermis (matrix, anchoring plaques (electron-dense structures), or LD)	• Intertwining between interstitial COL fibrils • Attaching the LD to the papillary dermis	• Cross-banded, fibrillar structures • A nonfibrillar COL composing of 3 identical α1 (VII) chains • Binding to: COL VII (NC1 domain) to COL IV (in LD) & Ln 5 (in LL) • Synthesized by both Ks & Fs COL

**Table 2:** Lists of different efforts to mimic the anatomical features of DEJ. Abbreviations: CI, Acid-soluble type I collagen; COL, Collagen; GAG, Glycosaminoglycan; FN, Fibronectin; H&E, Hematoxylin and eosin staining; NHKs, Neonatal primary human keratinocytes; NHFs, Neonatal primary human fibroblasts; HFs, Human dermal fibroblasts; HKs, human epidermal keratinocytes; Ks, Keratinocytes; Fs, Fibroblasts; SC, stem cell; PHEMA, Poly hydroxyethyl methacrylate; PLGA, Poly(d,l-lactide-co-glycolide); PEGDA, Polyethylene glycol diacrylate; PHBV, Poly(3-hydrroxybutyrate-*co*-3-hydroxyvalerate); PCL, Polycaprolactone; anti, Antibody; DAPI, 4′,6-diamidino-2-phenylindole; IHC, Immunohistochemistry; CEAs, Cultured epithelial autografts; TEWL, transepidermal water loss; IM, Immunodeficient mice; Assays: 1. Microtopographic analysis; 2. Cell type, culturing system and days; 3. Cellular investigations; 4. In vivo study; ↑, Improved or Higher; ↓, Decreased or Lower; =>, Resulted in.

	Biomaterial	Characterization Methods	Key Results, [Ref]
Photolithography	CI (microfabricated portion), COL–GAG (sponges) & FN (conjugated on surface)	1. H&E 2. NHKs; A/L interface; 3 or 7 days 3. Ki67 biomarker & IHC	Well-differentiated epidermal layers. ↑ Epithelialization for the narrow-width than the wider channels. Epithelialization like the natural process. A heterogeneous population of basal Ks. Providing an environment for SC niche. Detecting β1 integrin in µDERM channels [31].
	Same biomaterials (CI, COL-GAG & FN), but modified process by adding Fs (sponge) & reducing the CI’s thickness.	1. H&E. 2. NHKs & NHFs co-culture; A/L interface; 3 or 7 days 3. IHC	↑ Stratification in the graft regions containing microtopographies. ↑ Ks proliferative phenotype (in narrower channels). ↑ Synthesis of BM protein & Ln 332 (in wider channels). Detecting the β1^bri^p63 + Ks within the base of narrow channels & the corners of wider channels [13].
	PHEMA (mold, negative patterns) & PDMS (film; positive patterns) coated by Col type I *Static model*	1. SEM 2. NHKs for 2 days 3. IHC & DAPI	S1: the best pattern => recreated SCs distribution in the basal layer. Clusters of β1 integrin bright cells on the tips of topographies, particularly S1. Altering wavelength spacing & amplitude => changing pattern in the integrin-bright cells expression. No Ks differentiation on the tips [112].
		1. SEM 2. NHKs for 4 days 3. IHC, DAPI, Live/Dead & AFM (cell stiffness)	Expression & accumulation on the tips: β1 integrin bright cells. ↑ F-actin, Desmoglein 3 & ↓ MAL. ↑ Cell stiffness on the base. Rho-kinase activity => maintain adheren junctions. Rho kinase activity => differential stiffness of the cells. Forces exerted by cells on the slopes of the topographies => regulating SC patterning [113].
Laser	PLGA (membrane) coated by COL I & Polyimide (template) *Dynamic model*	1. SEM 2. NHKs for 2 days 3. IHC & DAPI	Formation of stratified basal sheets (β1 integrin-positive) & suprabasal. Differentiating cells (involucrin-positive). Clustering β1 integrin bright cells in the holes.YAP localization to the cell nucleus. At the edge of largest holes (topography 3) => DEJ formation by integrin bright cells with nuclear YAP [114].
Electrospinning	PHBV (scaffold) & PEGDA ( template by Stereolithography)	1. SEM 2 & 3. HKs; MTT (1, 3, & 7 days) & Live/dead (1, 3, & 7 days)	↑ Colonies formation retained within the microfeatures. Migration within the niche-like structures. Showing the typical K morphology [14].
	PCL (scaffold) & RS-F2-GPGR-04 (template by microtereolithography)	1. SEM & H&E 2 & 3. HFs & HKs co-culture; A/L interface; 1, 3, 6 (Resazurin), 10, & 12 days (in vitro skin model); IHC, DAPI & Lightsheet Microscopy	↑ Cell metabolic activity. HFs & HKs accumulation at the bottom of the microfeatures. COL IV & integrin β1expression at the bottom of the microfeatures. Pattern B: the best-promoting DEJ [115].
Laser structuring of electrospun mats	CI	1. H&E 2. HFs (5 days, on this day, the ridges generated) & HKs (20 days). 4. IM (grafting: 2 weeks & monitoring post-grafting: 4 weeks); Contraction evaluation; IHC & DAPI	↑ Epidermal barrier function (started at 2 weeks). ActiveFX & DeepFX: Dermal papilla-like generation. ↑ BM proteins levels (COL IV & rat anti-integrin alpha 6 (ITGA6)). ↑ Epidermal thickness & proliferative Ks. DeepFX grafts: the best-promoting DEJ, epidermal viability, & barrier function [33].
		1. SEM & H&E 2. HFs (5 days, ridges generation) & HKs (A-L; 3 & 11 days) 3. MTT, DAPI, IHC, & Quantitative gene expression 4. IM (grafting: 2 weeks & monitoring post-grafting: 4 weeks); Contraction evaluation; TEWL; PCR; IHC.	Organization of Ks in ridged samples. Formation discrete projections into the dermis. ↑ Ln expression. Expression of epidermal SC marker genes [15].
3D bioprinting	Silk fibroin & Gelatin	1. Light microscopy 2. HFs (3 days) & HKs (next 3 days); dual-layered 3D bioprinted constructs: A-L (21 days) 3. Live/dead, gene expression: RT-PCR (COL1A1, fibronectin (FN1) & Ln 1), total COL estimation (hydroxyproline) & IHC, genomic, & proteomic analysis	BM proteins expression => ↑ Mechanical strength. ↑ migration of cultured Ks. ↑ stretching of actin cytoskeleton & cell polarization (close the pores). ↑ integrins & focal adhesion => developing anchorage within pericellular niche. An akin FN distribution (similar to the native skin). Expression of ECM producing genes & differentiation proteins [116].
